# An Insight to Brain Targeting Utilizing Polymeric Nanoparticles: Effective Treatment Modalities for Neurological Disorders and Brain Tumor

**DOI:** 10.3389/fbioe.2022.788128

**Published:** 2022-02-03

**Authors:** Annu, Ali Sartaj, Zufika Qamar, Shadab Md, Nabil A. Alhakamy, Sanjula Baboota, Javed Ali

**Affiliations:** ^1^ Department of Pharmaceutics, School of Pharmaceutical Education and Research, New Delhi, India; ^2^ Department of Pharmaceutics, Faculty of Pharmacy, King Abdulaziz University, Jeddah, Saudi Arabia; ^3^ Center of Excellence for Drug Research and Pharmaceutical Industries, King Abdulaziz University, Jeddah, Saudi Arabia

**Keywords:** brain targeting, drug delivery, blood–brain barrier, central nervous system disorders, polymeric nanoparticles

## Abstract

The delivery of therapeutic molecules to the brain remains an unsolved problem to the researchers due to the existence of the blood–brain barrier (BBB), which halts the entry of unwanted substances to the brain. Central nervous system (CNS) disorders, mainly Parkinson’s disease, Alzheimer’s disease, schizophrenia, brain tumors, and stroke, are highly prevalent globally and are a growing concern for researchers due to restricting the delivery of pharmaceutical drugs to the brain. So effective treatment modalities are essential to combat the growing epidemic of CNS diseases. Recently, the growing attention in the field of nanotechnology has gained the faith of researchers for the delivery of therapeutics to the brain by targeting them to the specific target site. Polymeric nanoparticles (PNPs) emerge out to be an instrumental approach in drug targeting to the brain by overcoming the physiological barrier, biomedical barrier, and BBB. Preclinical discovery has shown the tremendous potential and versatility of PNPs in encapsulating several drugs and their targeting to the deepest regions of the brain, thus improving therapeutic intervention of CNS disorders. The current review will summarize advances in the development of PNPs for targeting therapeutics to the brain and the functional and molecular effects obtained in the preclinical model of most common CNS diseases. The advancement of PNPs in clinical practice and their prospect in brain targeting will also be discussed briefly.

## Introduction

The blood–brain barrier (BBB) works as a dynamic regulator in ion hemostasis and nutrient transport and a deterrent to harmful molecules (Fricker et al., 2014). It is composed of brain endothelial cells, astrocytes, and pericytes, which prevent the entry of all lipophilic molecules (above 500 Da) weight into the brain extracellular fluid. Unfortunately, the BBB prevents therapeutic molecules from reaching the brain, thus making a big obstruction for central nervous system (CNS) disorder treatment (Shakeri et al., 2020). From all global diseases, approximately 14% of the population is affected by CNS disorders (Saucier-Sawyer et al., 2015). About 1.5 million of the population is suffering from CNS disorders worldwide according to the World Health Organization (WHO) report (Annu Baboota and Ali, 2021). Conclusively, Parkinson’s disease (PD), Alzheimer’s disease (AD), brain tumors, schizophrenia, and stroke are major CNS disorders that are prevalent globally and are a growing concern for researchers, as the available treatment are unable to halt the progression of the disease. PD affects more than 10 million population worldwide among which nearly 1 million are in the United States, which is expected to be 1.2 million by 2030 (Bloem and Brundin, 2020). AD is the sixth leading cause of death, contributing 60%–70% of dementia cases globally (Hettiarachchi et al., 2019; Tiwari et al., 2019). According to the WHO report, about 50 million people are living with dementia, which is expected to be 152 million by 2050 (Agrawal et al., 2021a, Agrawal et al., 2021b). The annual incidence of malignant brain tumors is 3.7 and 2.6 per 100,000 for men and women, respectively, globally (Farmanfarma et al., 2019). In 2018, about 296,851 new cases of brain and other nervous system cancers were estimated according to global cancer statistics (Nehra et al., 20212021). According to the WHO statistics, 20 million people are affected worldwide with schizophrenia with 1.5 per 10,000 people of new cases (Fond et al., 2013; World Health Organization, 2019). About 87% of strokes are ischemic strokes. Over 13.7 million cases of strokes are reported each year globally among which one in four suffers from a stroke in their lifetime (Lindsay et al., 2019). Every year, a large number of patients are hospitalized due to the severe nature of CNS disorders, causing a substantial cost to the healthcare system. The approximate annual cost used for the treatment of CNS disorders in the United States alone is approximately $650 billion (Johnsen and Moos, 2016). Therefore, safe and effective treatment modalities that can traverse the BBB and deliver the therapeutic molecules directly to the affected areas of the brain need to be developed (Rehman et al., 2020).

Also, the development of new treatment for CNS disorders represents the largest market that was US$75.3 billion in 2010, $102.0 billion in 2015, and $35,497.3 million in 2018 and expected to grow ($62,786.2) million till 2026 according to a recent report by Fortune Business Insights (Neurodegenerative Disease, 2019; Shakeri et al., 2020).

The emergence of nanotechnology has gained much popularity as the drug delivery vehicle to the brain and brought the diversity of new feasibility in clinical practice and biological discovery. Particularly, nano-scaled carriers in drug delivery have revolutionized, allowing selective and targeted delivery of therapeutic carriers to organ, tissue, and cell-specific levels, also reducing the exposure of drugs to healthy tissues (Bhaskar et al., 2010). Polymeric nanoparticles (PNPs) opt out to be the ideal vehicles for therapeutic delivery to the brain owing to their unique physicochemical characteristics like high biocompatibility, biodegradability, and low toxicity. PNPs are colloidal carriers ranging from 10 to 100 nm in size and are composed of both natural and synthetic polymers. PNPs possess unique functionality, versatile surface properties, plasticity, and flexible nature (Annu Baboota and Ali, 2021).

Surface modification of PNPs with ligands like transferrin (Tf), apolipoprotein E, apolipoprotein B, apolipoprotein A, and antibodies have shown promising results in delivering the therapeutic carriers to the specific receptor-targeted site *via* the ligand-receptor mechanism. Ligand-mediated PNPs easily pass the BBB *via* receptor-mediated endocytosis, thus providing targeted delivery of therapeutics to the parenchyma of the brain. Also, PNPs can rapidly diffuse within the brain parenchyma when they are coated with a dense layer of polyethylene glycol (PEG) also termed as brain-penetrating NPs. PEG is a hydrophilic polymer that effectively reduces the adhesion between the particle and the charged components in the parenchyma of the brain (Nance et al., 2014). It was anticipated that the modification of NPs with targeting ligands will enhance the delivery of therapeutics to targeted or diseased tissues. However, the formation of protein corona on NP exposure to the bloodstream may highly alter the binding of the targeting ligand to its receptor (Xiao et al., 2021).

## Ligand Targeted Approach

**FIGURE 1:**
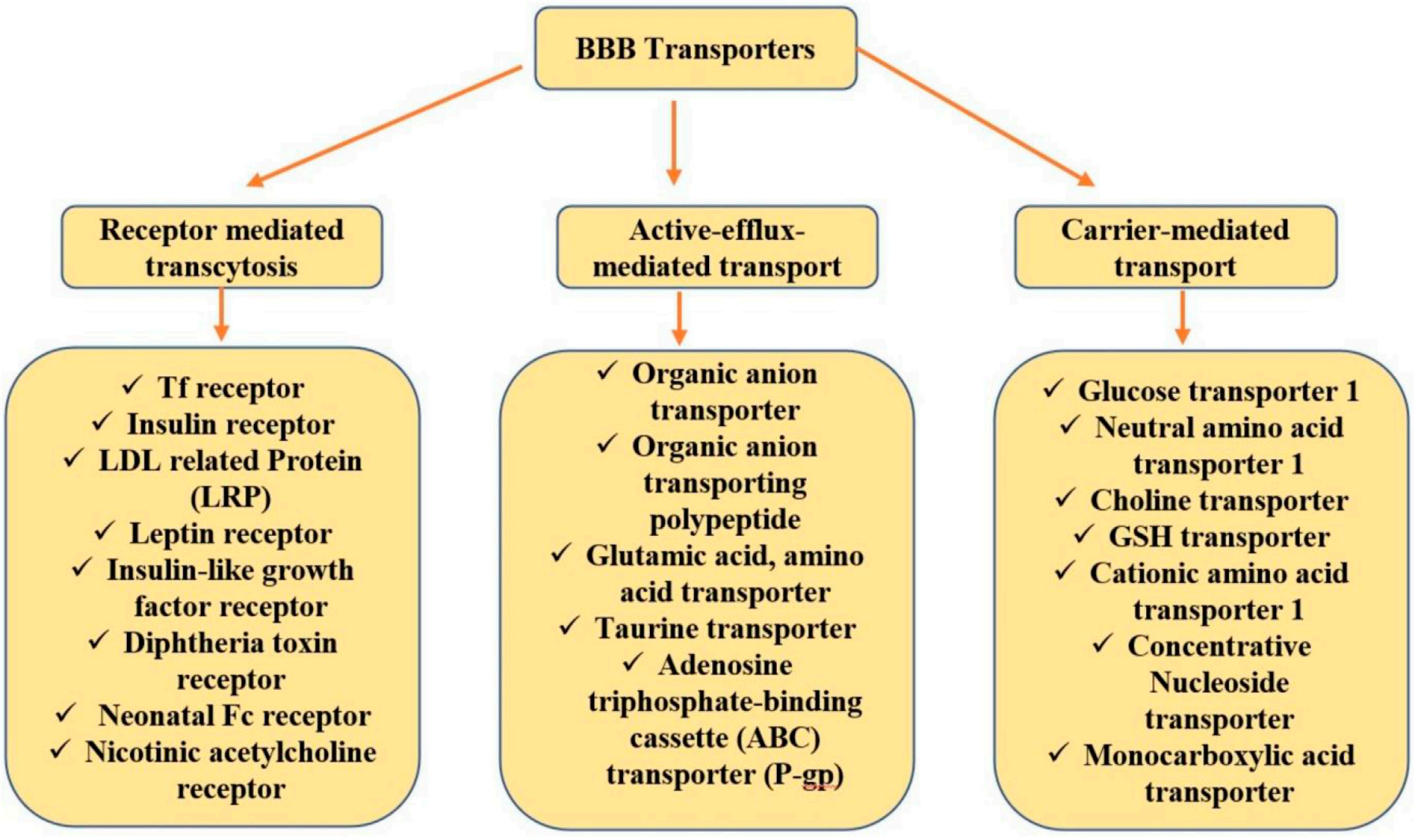
BBB transporters. BBB, blood–brain barrier.

Specifically, this is an active targeting approach where the ligand is either directly connected to the ligand surface or through a linker. Ligands can be segregated based on their mechanism of transcytosis. For conjugation with a drug, an ideal ligand should abide by a precise functional group as well as should have giant specificity towards the receptor (Nabi et al., 2018a). A brief view of ligands that bear enormous potential in drug delivery for CNS disorders is undermentioned and investigated based on the established literature. Figure 1 summarizes the various targeting or transporting approaches used for biological molecules delivery to the brain across the BBB.

### Transferrin Receptors

Earlier reports showed the expression of Tf receptor (TfR) in all nucleated cells and the BBB and overexpression in a variety of tumors (Nabi et al., 2018a). TfR is overexpressed in brain neurons, capillary endothelium cells, and choroid plexuses (Sundaram et al., 2009; Choudhari et al., 2021). Tf is a glycoprotein that binds to the iron in the blood and delivers it to the brain *via* TfR present on the BBB (Sharma et al., 2016a; Choudhari et al., 2021). The transport of Holo-Tf from blood to the brain and apo-Tf from the brain to blood is mediated by the TfR (Liu et al., 2013). Transport of Tf to the brain can be achieved *via* a receptor-mediated endocytosis mechanism. The cellular internalization of Tf-conjugated NPs to the targeted cells (brain neurons) can result in significant enhancement of drug therapeutic efficacy, thus reducing administered drug dose and dosing frequency. Tf conjugation provides site-specific delivery of the drugs, thus reducing drug-related adverse effects and peripheral drug toxicity (Liu et al., 2013; Nabi et al., 2018b). Some studies that target TfR for delivering drugs to the brain are mentioned below. Nabi and associates demonstrated the enhanced brain delivery of Tf-conjugated chitosan NPs in the management of neurodegenerative disorders (Nabi et al., 2020). Tf can be used for the transport of metallic particles; for example, gallium has widespread use as a targeted drug delivery carrier, that is, *via* the Tf pathway. Also, in DNA replication, the Tf role is reported (Vijayalakshmy et al., 2019). Tf (MAB) monoclonal antibody (OX26 or R17-217) or protein can be conjugated to the NP surface. Reportedly, the monoclonal antibody conjugated to Tf binds to different sites at TfRs in comparison to TfR protein; thus, there is a very low chance of interference of MAB-targeted Tf-NPs with endogenous Tf in circulation. Thus, to curtail the limitations of Tf as a drug delivery vehicle, TfR-targeted antibodies were used. i.e., 8D3 anti-mouse TfR monoclonal antibody (8D3), anti-rat TfR monoclonal antibody (OX26), and R17-217 (Sharma et al., 2016).

(OX26) Anti-TfR antibody is mainly used in brain targeting in the management of brain glioma and binds to the TfR epitope; thus, it does not compete with the natural binding site of Tf. It is basically an anti-rat TfR monoclonal antibody and does not bind to human TfR; thus, the clinical implication of this technology is impossible (Gaillard et al., 2005; Liu et al., 2013). It is transported to rats’ TfR *via* receptor-mediated transcytosis (Cano et al., 2020). OX26 is one of the most exploited antibodies of the Tf family due to its higher success in brain delivery (Nabi et al., 2018b). *In vitro* and *in vivo* studies revealed better brain delivery across the BBB of OX26-modified PEG liposomes with a lower tumor value and increased survival time (Yue et al., 2014).

R17-217 anti-TfR antibody also has the potential to enhance gene transfection and drug delivery, as it mediates NP transport across the BBB (Gao, 2016). It is a monoclonal antibody that binds specifically to TfRs *via* receptor-mediated endocytosis. A study conducted by Kang and associates summarized an increased penetration and uptake of docetaxel (DTX) liposomes co-modified with muscone and R17-217 into tumor spheroids. Also, *in vivo* results reported increased brain targeting and survival rate in tumor-bearing nude mice (Kang et al., 2020).

Apo-Tf is related to the Tf family and possesses Tf-like characteristics when it is unbound to iron. It is a single-chain glycoprotein that exists in the plasma as mono or differic Tf, having a high affinity towards ferric iron with two nonidentical iron-binding sites. Choi and Park developed DTX nanocrystals and modified them with human apo-Tf to improve the cytotoxicity and cellular uptake of DTX (Choi and Park, 2017). Infusion of Apo-Tf might minimize the oxidative damage by binding to the released iron during radiotherapy (Vijayalakshmy et al., 2019). It was found from the literature that administration of Tf *via* nasal route is a fruitful approach that provides protective action to neurons in hypoxic–ischemic management; the study was implicated on mouse and rat models.

Lactoferrin (Lf) a cationic glycoprotein (iron-bounded) that belongs to the Tf family and exhibited higher activity in comparison to OX26 and Tf for transferring active therapeutics across the BBB (Gao, 2016). Lf exhibits antibacterial, antiviral, antimicrobial, and antitumor biological functions. It crosses the BBB *via* transcytosis pathways within endothelial cells of the brain capillary (Tomitaka et al., 2015). Lf-modified NPs have been widely used for targeting brain glioma. Li and associates developed Lf-modified PEG–polylactide (PEG-PLA) NPs of shikonin to target the glioma cells *via* receptor-mediated endocytosis. Modified NPs revealed sustained release of more than 72 h and higher brain concentrations *in vivo* than free shikonin NPs and shikonin (Li et al., 2017). The report suggested that in targeting brain glioma using magnetic particle imaging, Lf-modified iron oxide NPs can be used as tracers (Tomitaka et al., 2015).

Insulin receptor antibody exhibits very high BBB-penetrating efficiency compared to TfR antibodies. The insulin receptor is abundantly present in the brain (hypothalamus, hippocampus, cerebral cortex, and olfactory bulb). In CNS, these receptors have effective action in neuronal survival, homeostasis, and reproductive endocrinology (Choudhari et al., 2021). Insulin is an endogenous protein that is transported *via* receptor-mediated transcytosis to the brain and specifically acts on insulin receptors (Cano et al., 2020). The transport of insulin across the brain parenchyma can be influenced or increased by pro-inflammatory factors like tumor necrosis factor and interleukin-1 (Pomytkin et al., 2018).

### Cell-Penetrating Peptides

Cell-penetrating peptides (CPPs) are amphipathic or small cationic cargo having the ability to transport molecular molecules like (oligonucleotides, peptides, proteins, nanoparticles (NPs), liposomes, etc.) to the innermost cells. CPPs can cross the cell membrane *via* energy-independent or receptor-mediated processed, and without compromising their bioavailability, they efficiently internalize the associated biomolecules to the cells. They have been exploited widely to establish their efficiency in the management of brain-related diseases. Various kinds of CPPs used in the medical domain are penetratin, transactivator of transcription peptide (TAT), and poly-l-arginine (Sharma et al., 2016a; Nabi et al., 2018a). Currently, about 1,850 kinds of CPP sequences are available on the CPP site 2.0 database. The exact mechanism of CPP cellular uptake remains controversial; however, the general mechanism for CPP uptake is considered to be endocytosis or non-endocytosis direct translocation (Xie et al., 2020). They can transport therapeutic agents across the BBB through adsorptive-mediated transcytosis and demonstrated BBB translocation in submicromolar concentrations without generating cytotoxic effects (Sharma et al., 2016b; Xie et al., 2020). A cell-penetrating mitochondrion-targeting peptide was designed computationally to transduce human metallothionein 1A (hMT1A) (antioxidant protein) into mitochondria, which in the PD mouse model rescued dopaminergic neuronal degeneration and movement impairment (Kang et al., 2018).

### Apolipoprotein (E)

It is a specific receptor that is present on the BBB and helps in selective and specific delivery of drug lipoprotein complex. It is related to high-density lipoprotein, very-low-density lipoprotein, and specifically a 34-kDa protein class that helps in the transportation of lipids and cholesterol across the BBB (Bu, 2009). Apo-E is transported *via* the receptor-mediated endocytosis mechanism to the endothelial low-density lipoprotein (LDL) receptor (Cano et al., 2020). Human Apo-E differs both functionally and structurally from mouse Apo-E. It modulates multiple mechanistic pathways (e.g., cholesterol/lipid homeostasis, glucose metabolism, synaptic function, neurogenesis, neuroinflammation, and amyloid-β aggregation) in CNS, which affects cognition (Tai et al., 2016). A 1.8-fold increase in cellular uptake of palmitate solid lipid NPs (SLNs) modified with APO-E and a 1.9-fold increase in DSPE-Apo-E-modified SLNs were reported by Neves and associates (Neves et al., 2017). A significant enhancement in brain uptake *via* Apo-E NPs was observed by Hartl and associates (Hartl et al., 2021).

### Low-Density Lipoprotein-Related Protein

It is categorized as LRP1, which is present on the BBB and brain, whereas LRP2 protein is specifically found only in the brain. *In vitro* BBB model and *in situ* bovine brain perfusion of Aprotinin (LRP) ligand showed higher transcytosis across capillary endothelial cells of the brain (Harilal et al., 2020). Both LRPs are closely or structurally related to the gene family of the LDL receptor. They are multiligand scavengers and multifunctional and signaling receptors (Gaillard et al., 2005). They are transported to the endothelial LDL receptor *via* receptor-mediated endocytosis (Cano et al., 2020).

### Anti-Human Epidermal Growth Factor Receptor Antibody

Epidermal growth factor receptor (EGFR) is a transmembrane glycoprotein receptor whose extracellular domain acts as a receptor, while the intracellular domain acts as tyrosine kinase with a high affinity towards EGF (Nabi et al., 2018a). In cancer cells, they are expressed highly, and more than 40% are expressed in glioblastoma (GBM), indicating their role in the pathogenesis of GBM (Mortensen et al., 2013). About 25% of GBM is characterized by EGRFR mutant expressions that are defective of extracellular ligand-binding domain. In preclinical and clinical trials, lapatinib, gefitinib, and erlotinib have been tested for malignant glioma treatment (Ferraris et al., 2020a). For the treatment of head, neck, and metastatic colorectal cancers, cetuximab is an EFR-targeting antibody approved by the Food and Drug Administration (FDA) (Desnoyers et al., 2013). Mouse anti-human EGFR nimotuzumab antibodies in clinical trials were employed for recurrent high-grade glioma patients (Ferraris et al., 2020a).

### Other Transporters

#### Glucose Transporters

GLUT is majorly expressed at the brain capillary luminal surface with choroid plexus and can be efficiently used for the delivery of therapeutic biomolecules to the brain. Cholesterol glucosyl derivative was formulated by Quin and associates to enhance the therapeutic efficacy of drugs in the brain (Qin et al., 2010).

#### Glutathione Transporters

Glutathione (GSH) is an endogenous tripeptide with antioxidant nature and expressed highly on the BBB. Previous literature proved that GSH has a profound ability to enhance brain uptakes when they are conjugated to liposomes (Gaillard et al., 2012). Thus, they can also be explored for the delivery of NPs into the brain.

#### Choline Transporters

These are precursors of neurotransmitters and acetylcholine; that is why they are tremendously expressed in the brain. Choline does not have the flexibility to be modified, so quaternary ammonium compounds were researched that can bind choline receptors and act as choline transporters (Gao, 2016).

### Challenges Associated With Targeted Approach

As the name suggests, the “targeting approach” reflects the ability of the drug delivery system to target the specific site to achieve therapeutic effects. But the plethora of several features influencing the effectiveness of the targeting systems hinders the achievement of the therapeutic efficacy of these systems. Insufficient targets, in addition to the choice of the target with different alignments to the interruption necessities, cause difficulty in this process. Moreover, for sufficient uptake of the drug by cells, choosing suitable target epitopes is required to regulate the approachability of attaching drug conjugates with bulkier drug carriers. However, designing of these targeting systems must acclimatize numerous physiological variables including the flow of blood, status of the disease, and the architecture of the tissues by lodging physicochemical constraints including the structure of the nanocarriers, composition of the carriers of the drugs, and how to put them into work (Muro, 2012). The applications of the targeted delivery system are much more associated with some hurdles to the effectual delivery of the drug. There is an occurrence of low bioavailability of the drugs at the target site when delivered using these carriers; these systems are prone to opsonization and thereby suffer from unwanted reticuloendothelial system (RES) uptake directly affecting the drug efficacy. In addition to these, these carriers voluntarily erupt from circulation *via* vascular gaps or any defect credited in process angiogenesis that is characteristic of tumor sites (Cukierman and Khan, 2010).

### Advantages Associated With Targeted Approach

Apart from the challenges associated with the targeted drug delivery systems, there are some benefits that these delivery systems offer including maintenance of the drug level in the plasma and tissues thereby evading any harmful effect of drugs on the healthy cells (Cukierman and Khan, 2010; Pattni and Torchilin, 2015). Moreover, these systems are preferred much for the drugs belonging to BCS class IV. A targeted drug delivery system helps in enhancing the absorption of the drug or combination of the drugs at the diseased site. In comparison with conventional drugs, this system is highly specific and desirable, thus increasing the therapeutic profile of the drugs (Rani and Paliwal, 2014).

### Advantages of Metal Oxide-Based Nanoparticles

These NPs or nanomedicine have tremendous properties like antimicrobial activity, anti-insecticidal activity, and nontoxicity; hence they can be used in the diagnosis of life-threatening diseases. Cerium NPs can help in combating AD and cancer, but their use is limited due to the lack of stability in living organisms (Singh et al., 2021). Similarly, iron oxide NPs showed great properties in GBM treatment (Kievit and Zhang, 2011; Tomitaka et al., 2015). Cobalt NPs have good magnetic, optical, mechanical, and chemical properties and are successfully used in MRI. Moreover, cerium oxide NPs have excellent antioxidant properties and can be used in the treatment of neurodegenerative disorders and ischemic stroke/cerebral stroke (Rzigalinski et al., 2006; Singh et al., 2021). Selenium was also reported to reduce the risk of neurodegenerative diseases in various animal models. However, there is a lot of research needed to develop metal and metal oxide NPs to treat CNS disorders efficiently (Singh et al., 2021).

## Polymeric Nanoparticles for Brain Delivery

**FIGURE 2:**
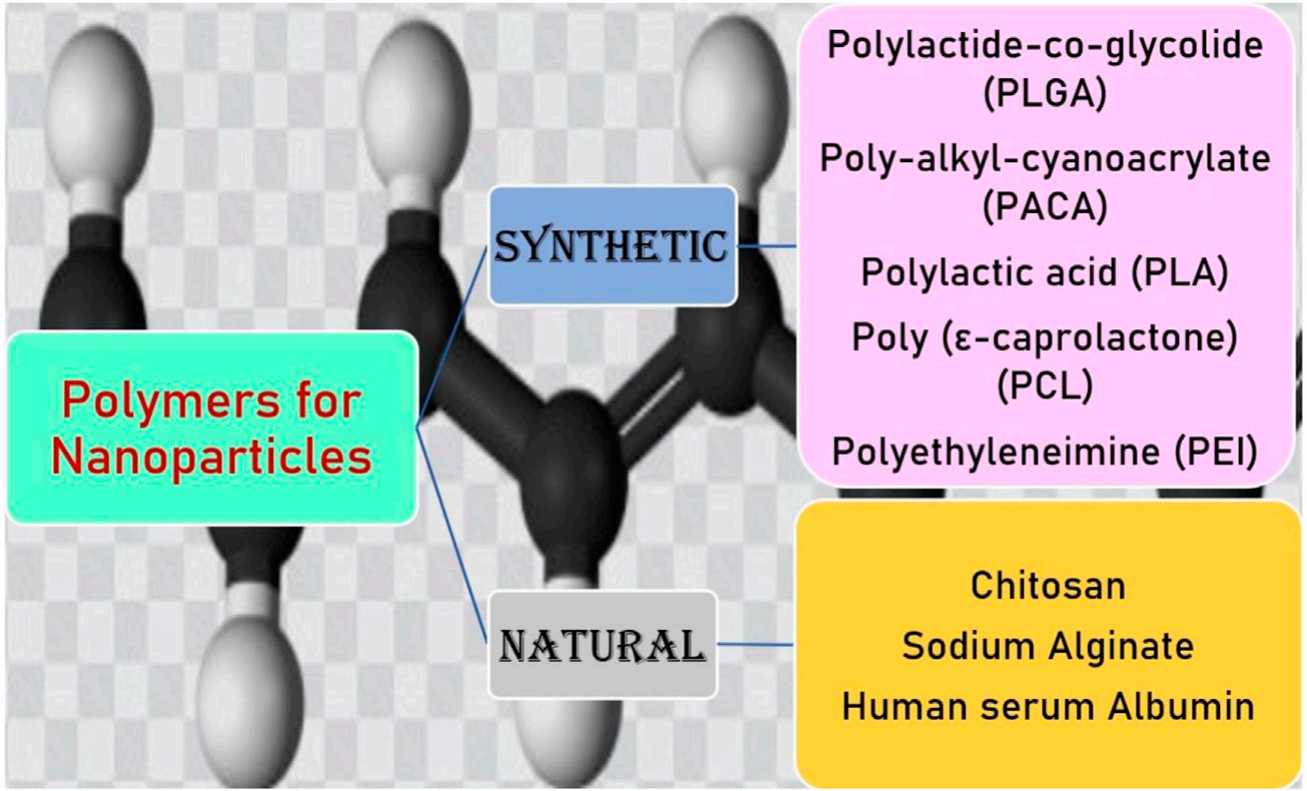
Types of polymers used for NP preparation for brain targeting. NP, nanoparticle.

PNPs are versatile drug delivery systems, which exhibit distinct shapes and physiochemical properties and could be widely used for the delivery of diverse drugs (Zhong et al., 2014). PNPs are easy to prepare and scale up, have high stability *in vivo* and during storage, and allow sustained drug release; thus, they come out to be the most promising carriers in improving drug delivery across the BBB (Martín-Banderas et al., 2011). While developing the PNPs, utmost care should be taken in selecting the appropriate polymer in terms of its safety and efficacy. The selected polymer must be biodegradable, biocompatible, nontoxic, non-immunogenic, and inexpensive. In the preparation of NPs for brain targeting, synthetic and natural polymers are used. Natural polymers included chitosan, human serum albumin, sodium alginate (Shakeri et al., 2020). Chitosan is the most commonly used polymer for brain targeting, as it exhibited the greatest bioadhesive and biocompatible properties (Agrawal et al., 2020). Chitosan is a cationic polymer that has a tendency to be easily attracted to the endothelial cells, which helps in the enhancement in their brain delivery and cellular absorption (Peniche and Peniche, 2011). Moreover, the modification of chitosan with specifically targeted ligands can increase the cellular uptake of NPs *via* receptor-mediated endocytosis. The most commonly used synthetic polymers for brain delivery of NPs are poly-alkyl-cyanoacrylate (PACA), polylactide-*co*-glycolide (PLGA), PEG, poly-ε-caprolactone (PCL), and PLA. These polymers are approved by the FDA for pharmaceutical purposes (Tosi et al., 2008; Severino et al., 2019; Zhao et al., 2020). NPs made of PLGA and PLA biodegradable polymers for pharmaceutical purposes are approved by the USFDA and show successful uptake with insulin, Tf, and LDL receptor (Onyema et al., 2021) (Onyema et al., 2021). PLGA is a copolymer approved by the USFDA and has nontoxic, non-immunogenic, biodegradable, and biocompatible characteristics. PLGA crosses the BBB and provides sustained release of the drug. PLGA-PEG-PLGA triblock with polysorbate 80 coating has been engineered to transport the drug through the BBB (Sardoiwala et al., 2018). PACA NPs for the treatment of CNS disorders are first studied by Couvreur and co-workers. PACA NPs have a higher half-life, drug bioavailability, and low toxicity and can overcome multidrug resistance (Shakeri et al., 2020; Zhang et al., 2021). PACA NPs are modified with PEG or polysorbate 80 to enhance their ability to penetrate the BBB (Zhang et al., 2021). PCL is an FDA-approved biodegradable polyester used in implants, sutures, and drug delivery. They have been used for the drug delivery to neurological disorders such as PEG-PCL micelles functionalized with peptide, which significantly enhance the transport and accumulation of NPs in an *in vivo* model of intracranial glioma tumor. The major limitations of these polymers are their lower degradation rate (Zhang et al., 2021). PEG has excellent biocompatibility and is widely used in drug delivery and biomedical and tissue engineering. PEG increases the shelf-life of drug molecules and is used in the brain to target various drug molecules. In an aqueous solution, they form micelles due to their self-organization ability (Kumar et al., 2020). Various kinds of polymers used for PNPs delivery to the brain are described in Figure 2.

Further, to facilitate drug delivery, PNP surfaces can be modified to escape them from the RES. For crossing the BBB and targeting the CNS *via* PNPs, different strategies have been explored, as mentioned below (Tosi et al., 2008).

❖Surface charge-based application in which mainly the positive surface charge of PNPs will be utilized to produce an adsorption-mediated transcytosis
❖Utilization of surfactants like Tween 80 (T80) and poloxamer as a coating agent, which could be coated or linked to PNP surfaces to target the BBB
❖Surface modification of PNPs using PEG to provide long-circulating characteristics and BBB-crossing ability to PNPs
❖Ligand conjugation with specific proteins, antibodies, peptides, etc., on PNP surfaces to enhance brain targeting *via* effective movement across the BBB crossing
❖Utilization of magnetic field for driving NPs to the BBB target site

**FIGURE 3:**
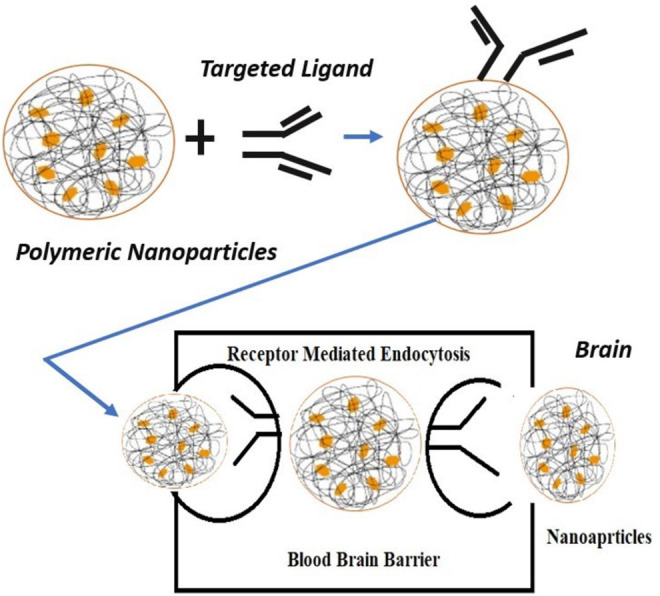
Mechanism of ligand-targeted polymeric nanoparticles transport through the blood–brain barrier.

For TfRs and glioma cells, which are upregulated transporters and receptors, the substrates or ligands have been conjugated to NP surfaces so that they can specifically bind to the overexpressed targets of diseased cells and trigger them *via* endocytosis, which is described in Figure 3 (Bloem and Brundin, 2020).

Various PNPs developed for targeting therapeutics to the brain for the treatment of neurological disorders like PD, AD, psychotic disorders, brain tumors, and stroke will be further discussed in this review, and the molecular and functional effects obtained in the preclinical model will be outlined.

### Effect of Nanoparticle Size and Shape in Central Nervous System Delivery

To reach the target site, extravasation is the first step for NPs in circulation, which can be altered by NP size and shape, as smaller NPs more easily cross the capillary walls than larger NPs. NPs show size-dependent distribution across the organs (Mitchell et al., 2021). If only considering the size, then studies also supported that NPs within 100 and 200 nm in size are more suitable due to their prolonged blood circulation and low uptake by the mononuclear phagocyte system, and also they have the potential to cross the BBB (Kulkarni and Feng, 2013; Wei et al., 2018). Single-walled carbon nanotubes with 30-nm size showed both higher nuclear and cellular internalization than 50 nm (Donkor and Tang, 2014). Most studies performed *in vitro* demonstrate that NPs with the size range of 10–60 nm have higher cellular uptake in nonphagocytic cells irrespective of their surface charge and composition (Hoshyar et al., 2016). The shape of NPs has a significant influence on their biodistribution, clearance, and cellular internalization. Studies demonstrated that NPs with worm-shape exhibit very long circulation than other shapes (Wei et al., 2018). The report suggested higher cell adhesion efficiency of elongated NPs due to their higher surface area, which helps in multivalent interaction of particles with the cell surface, whereas spherical NPs have a limited number of binding sites and smaller contact area due to their curved shape (Agarwal et al., 2013). *In vitro* and *in vivo* studies demonstrated higher efficiency and affinity of rod-shaped NPs to endothelial cells. Also, ellipsoidal NPs demonstrate lower cellular uptake in comparison to spherical NPs (Salatin et al., 2015). Nowak and associates in their studies showed size-dependent transport of NPs through endothelial cells. It was found that the particles with 200-nm spheres have 3-fold higher permeation than 100-nm spheres, and 100- and 10-fold lower permeation of 500-nm NPs than 200- and 100-nm spheres, respectively. Nontargeted rods showed lower adhesion on the synthetic surface than spheres. Also, the data revealed that rods shaped NPs have higher transport across the brain endothelium than spheres. Stiff particles showed higher transport than their soft counterparts (Nowak et al., 2019).

### Limitations and Challenges of Polymeric Nanoparticles in Brain Delivery

NPs have limited application in brain tumors, as they have insufficient retention and accumulation within the tumors due to leaky vasculature and restricted target specificity, as the exclusivity of known receptors is very less in brain tumors. Also, the inability of NPs to traverse the BBB in brain glioma is a limitation in delivering the drugs to the brain (Jain, 2012; Masserini, 2013; Belykh et al., 2020). Maintaining the safety, efficacy, and tolerability of NPs into the brain is a major challenge (Cano et al., 2020). The most prominent challenges linked to NPs are their industrial scale-up and complex regulatory approval. However, to evaluate the therapeutic potential of NPs in neurodegenerative diseases, clinical trials have been started (El-Say and El-Sawy, 2017; Cano et al., 2020). The particle size and charge of NPs affect the brain delivery of NPs, as particles with 100–200 nm in size are considered to be optimum for brain delivery (Shakeri et al., 2020). So the development of optimum size NPs is also a challenging task. In the extracellular matrix, the diffusion of NPs is limited due to their large size (Barua and Mitragotri, 2014).

### Application of Polymeric Nanoparticles in Brain Delivery

**FIGURE 4:**
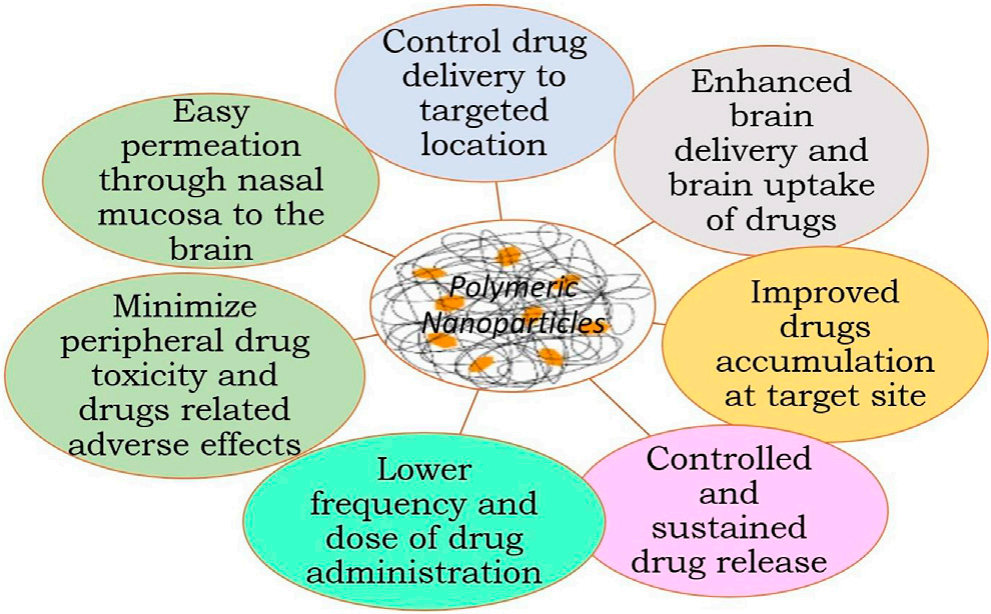
Application of polymeric NPs in brain delivery. NPs, nanoparticles.

Figure 4 summarizes the application of NPs in brain delivery (Nair et al., 2016; El-Say and El-Sawy, 2017; Ferraris et al., 2020b; Cano et al., 2020).

✓ They can be easily transported across the BBB without any damage to the BBB
✓ NPs can deliver the small molecules, nucleic acid, and protein to the brain
✓ Provide slow or sustained drug release, thus decreasing frequent drug dosing and drug dose
✓ Provide specific brain targeting after surface modification of NPs with appropriate targeting carrier
✓ Higher drug therapeutic efficacy
✓ Reduce drug-related adverse effects and peripheral drug toxicity
✓ Increase half-life of drugs
✓ Impart stability and longer activity to volatile active agents
✓ Controlled delivery

### Modification of Nanoparticles With Chitosan to Enhance Brain Delivery

**FIGURE 5:**
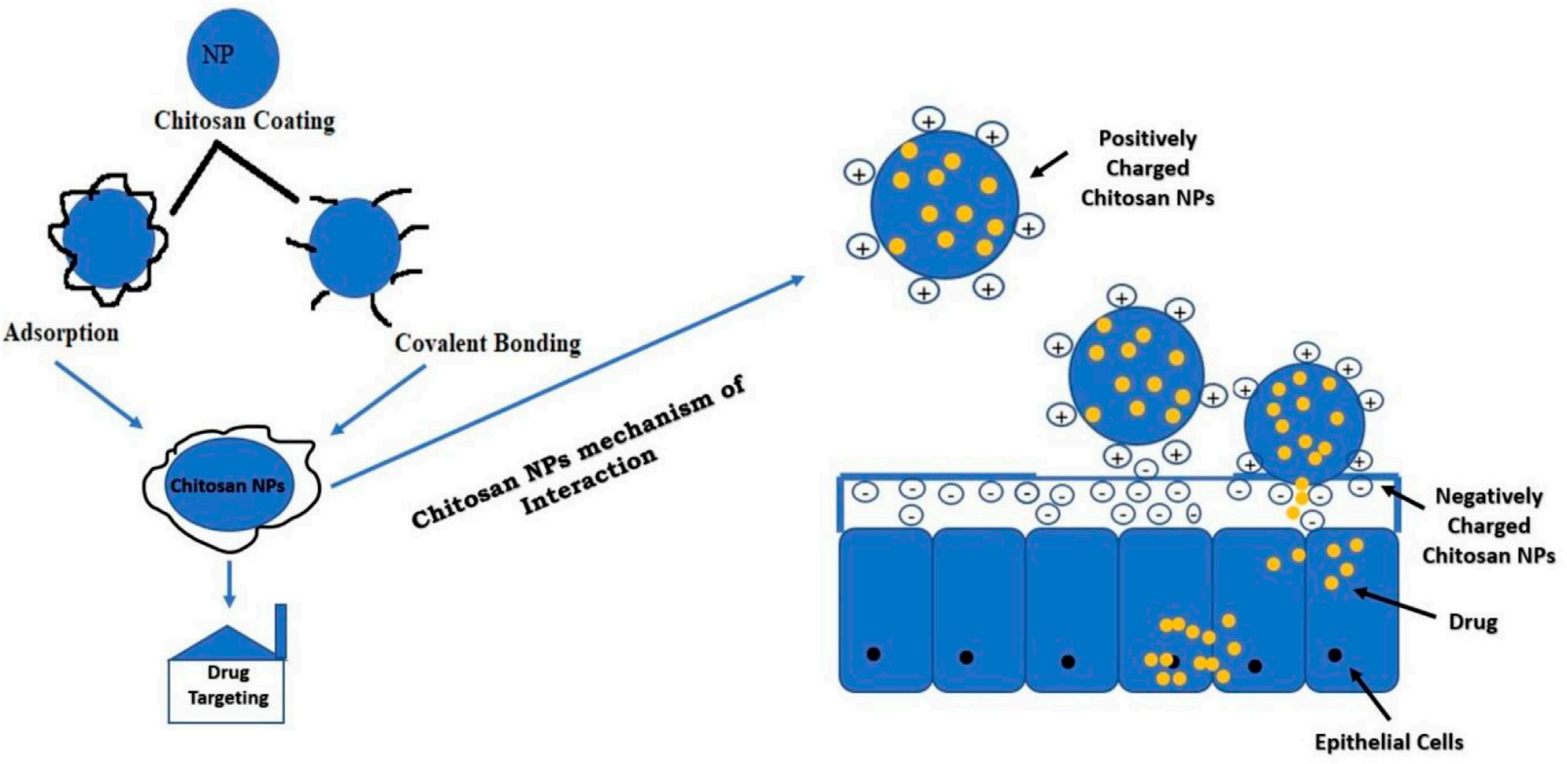
Strategies of NP modification with chitosan for drug delivery. NP, nanoparticle.

The amine-NH_2_ and hydroxyl-OH group present in the chitosan are active sites for modification. The modification will impart solubility, stability, and bioadhesion to the NPs. The most commonly employed techniques for the preparation of chitosan polymers are blending, curing, and graft co-polymerization. Positively charged chitosan NPs interact with negatively charged mucous membrane and release the drug (Mohammed et al., 2017). Adsorption of chitosan is a physiochemical phenomenon in which the polymeric material chains are deposited on the NP surface. The interaction or covalent bonding between functional groups of chitosan and NPs can be obtained *via* different reactions before or after NP preparation, which depends on the chemical properties of the materials and the method of synthesis. The modification of NPs with chitosan will enhance its brain delivery (Prado-Audelo et al., 2020). Figure 5 summarizes the strategies of NP modification with chitosan for drug delivery.

## Polymeric Nanoparticles in Treatment of Neurological Disorders and Brain Tumors

### Polymeric Nanoparticles in Parkinson’s Disease

PD is a growing and appalling neurodegenerative condition of the brain or CNS. It is mostly identified by dopaminergic neuron degeneration in the nigrostriatal bundle and midbrain region, which connects the pars compacta of the substantia nigra along with the dorsal striatum (Paul and Yadav, 2020). Poor balance, reduced motor control, tremors, and muscle stiffness are the primary symptoms of PD. The medication (levodopa, MAO-B inhibitors, dopamine, etc.) used for PD treatment aims to reduce the dopaminergic deficit. Though these medications dominate the treatment practice, the conventional dosage forms of these drugs suffer from enzymatic degradation, peripheral side effects, and low therapeutic efficacy. So novel therapies such as PNPs have gained much attention by the researchers for the treatment of PD patients, which provide the target-specific delivery, enhance therapeutic drug efficacy, and omit drug peripheral toxicity (Gan et al., 2019; Paul and Yadav, 2020). Various PNP-based formulations developed by the researchers for the treatment of PD are mentioned hereafter.

**FIGURE 6:**
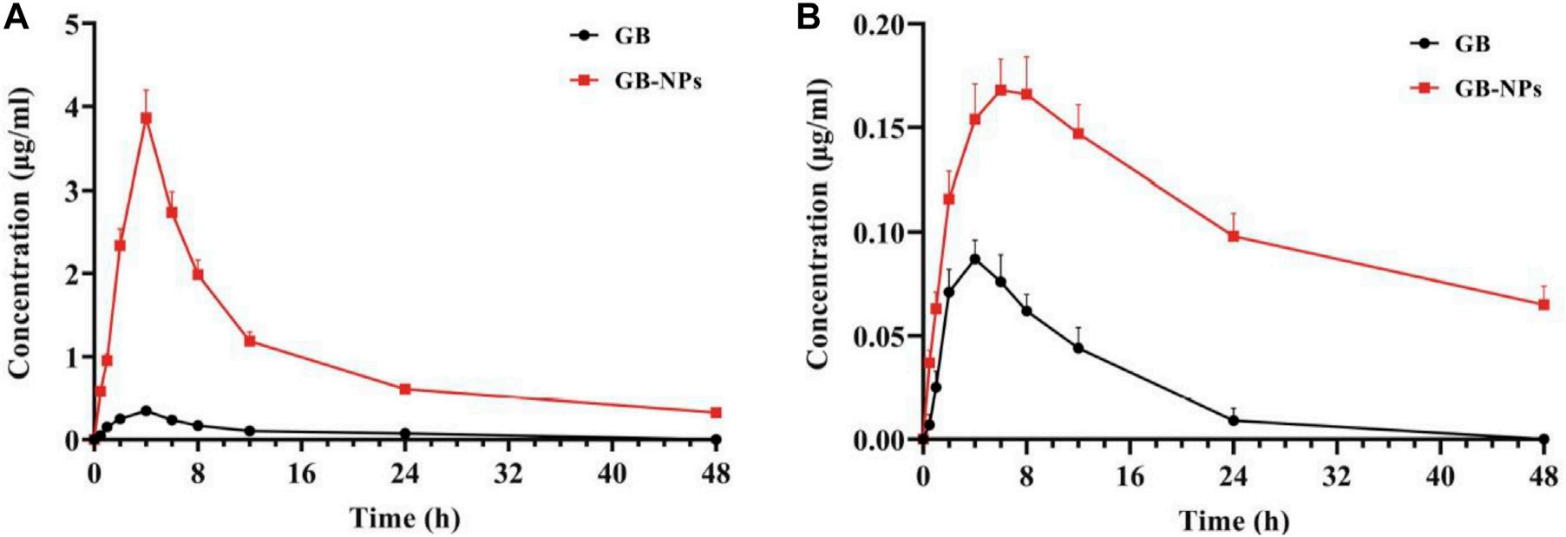
Plasma and brain pharmacokinetic profiles of GB and GB-NPs in rats. **(A)** Plasma concentration–time profile (*n* = 6). **(B)** Brain concentration–time profile (*n* = 4). Reproduced from open-access Journal under the term of Creative Commons Attribution (Zhao et al., 2020). GB, ginkgolide B; NPs, nanoparticles.

The poor oral bioavailability of ginkgolide B (GB) limits its clinical efficacy in PD treatment despite having good neuroprotective action. PEG-PCL NPs of GB were engineered by antisolvent precipitation and stabilization-based technique. PEG-PCL (20 mg/ml of acetone) and 2 ml of GB were mixed with 10 ml of dH_2_O having F68 (0.5 mg/ml) stabilizer with constant stirring at 1,000 rpm and room temperature. Similarly, C-6-NPs were prepared by substituting GB for C6 under dark conditions. The study conducted in Madin–Darby canine kidney cells depicted that the prepared NPs were effectively taken by the body *via* multiple nonspecific mechanisms including clathrin-mediated endocytosis, micropinocytosis, and caveolae/lipid raft-mediated endocytosis. NPs of GB showed the ability to transport across chorion, the BBB, gastrointestinal (GI) barrier, and blood–retinal barrier (BRB) in a zebrafish model. Pharmacokinetics data revealed the enhanced uptakes (11.03-fold) of GB in rats’ brains and plasma after oral administration as compared to free GB. Figure 6 shows the pharmacokinetic profiles of free GB and NPs of GB in the rat brain and plasma. Also, GB-NPs demonstrate higher therapeutic efficacy and reduced GB toxicity in the murine PD model as compared to free GB (Zhao et al., 2020).

*Author’s opinion*: Thus, the above study demonstrates that PEG-coated PNPs might be potential vehicles for targeting therapeutic active molecules to the brain to treat PD.

**FIGURE 7:**
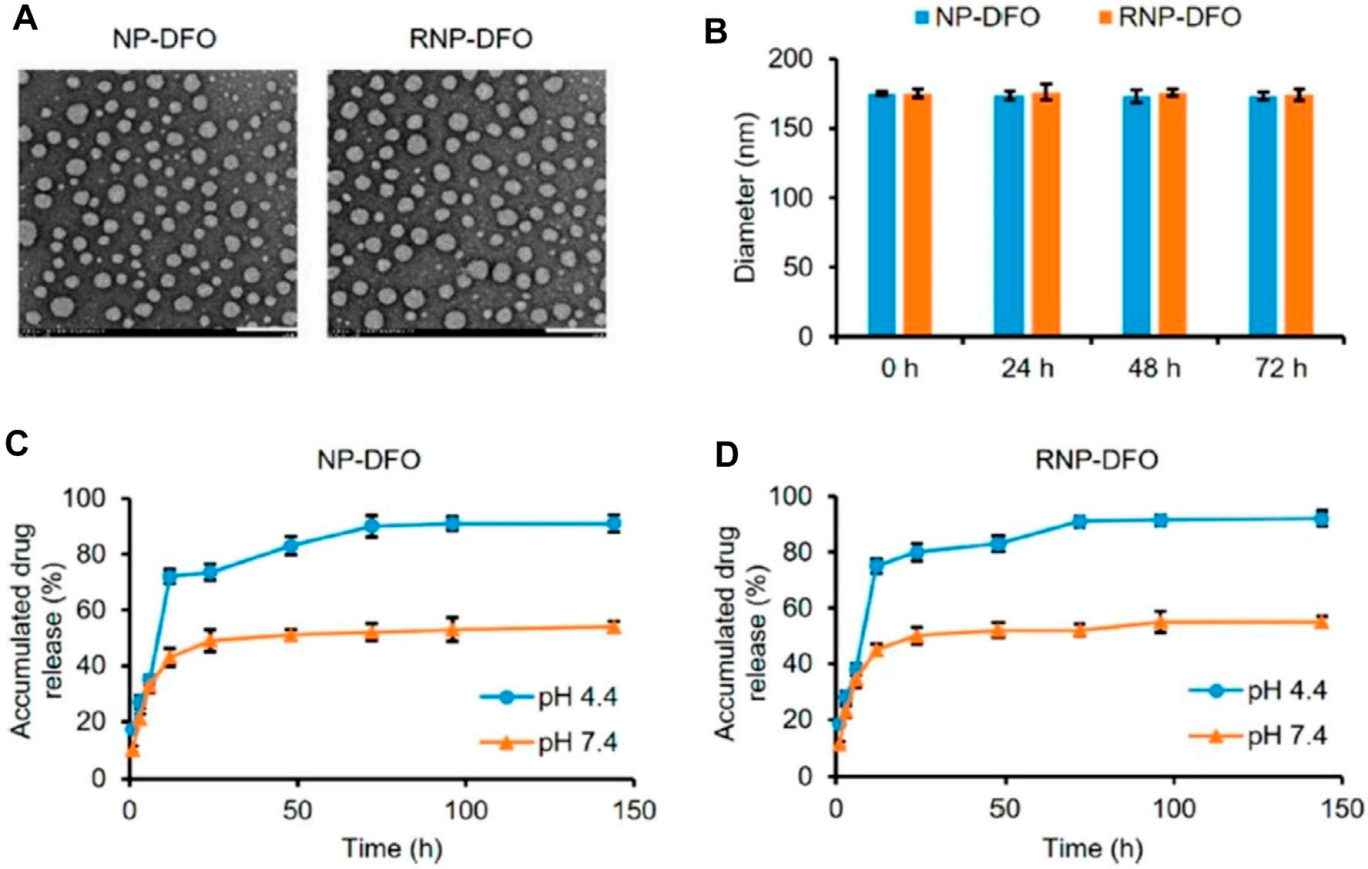
Characterization of DFO-loaded nanoparticles. **(A)** Photomicrographs of NP-DFO and RNP-DFO taken through TEM depict their smooth spherical morphology. Scale bar = 200 nm. **(B)** Diameter of NP-DFO and RNP-DFO before (0 h) and after being incubated in PBS (pH 7.4) for different periods as determined by DLS. No significant size change occurred in 72 h, suggesting that the nanoparticles are stable. Data are presented as mean ± SD from three independent experiments. **(C, D)** Release kinetics of DFO under mimic physiological conditions from NP-DFO **(C)** and RNP-DFO **(D)** at pH 4.4 and 7.4, respectively. Reprinted with permission from You et al., 2018 (*ACS Nano*). DFO, deferoxamine; NP, nanoparticle; TEM, transmission electron microscopy; PBS, phosphate-buffered saline; DLS, dynamic light scattering.

In PD, the excess deposition of iron may often result in dopaminergic neuron necrosis and oxidative stress. To decrease oxidative stress in PD striatum and substantia nigra of mice, You and associates used deferoxamine (DFO) polymeric NPs modified with rabies virus glycoprotein (RVG)29 as brain targeting peptide for intracerebral delivery of DFO. RVG29- and DFO-loaded NPs exhibited mean diameter of 168.8 ± 1.9, −27.40 ± 0.71 zeta potential, uniform and smooth morphology represented by transmission electron microscopy (TEM), and about 75% of *in vitro* drug release in 24 h (Figure 7). The study revealed the penetrations of NPs through the BBB *via* receptor-mediated endocytosis mechanism. NPs showed a significant decrease in oxidative stress and iron content and reversal in neurobehavioral deficits without any toxic effects to the brain and other organs in the PD mouse model. The study holds promising results for DFO delivery to the brain and utilizing iron chelation therapy for the treatment of PD (You et al., 2018).

### Neuroinflammation in Parkinson’s Disease

Neuroinflammation occurs in the subventricular zone where the proliferation of neural stem cells happens and repeats into new neurons and astrocytes. PNPs showed promising potential in boosting neuroprotection, which is studied by Gan and his associates. Surface-engineered PNPs of RVG29-loaded with microRNA (miR)-124 were designed by Gan and co-workers to improve neuroinflammation in PD. Polymeric NPs were prepared using a double-emulsion technique, and maleimide–thiol interaction was used for RVG29 conjugation onto the NP surface. *In vitro* drug release data demonstrate sustained miRNA release up to 60 h with approximately 40% of released in 24 h while ∼70% release at 60 h. RVG29-conjugated miRNA-NPs showed higher permeability *in vitro* BBB model in comparison to nontargeted NPs. MTT assay performed in BV 2 cells showed no toxicity of blank NPs for <100 μg/ml concentrations, indicating the safety of NPs. A marked decrease in cell viability after treatment with 200 μg/ml of placebo NPs was observed. Results reported a significant increment of inducible nitric oxide synthase (iNOS), tumor necrosis factor-alpha (TNF-α), cytokines (interleukin-6), and mRNA levels after lipopolysaccharide administration. However, the levels of mRNA in the miR-NP treated control group and the abovementioned cytokines were found almost equal, suggesting miR-124 exposure. Also, miR-NPs showed a significant reduction in expression of P-P65 and MEKK3 protein kinase. The studies indicated that miR-124 could target nuclear factor kappa B cell (NF-κb) pathways and MEKK3. mi-RNA-NPs results in the downregulation of MEKK3 expression in animal studies with ca. threefold decrease in cell apoptosis. The research revealed that mi-RNA-NP exhibits the potential to boost neuroprotection by inhibiting pro-inflammatory signaling (Gan et al., 2019).

### Polymeric Nanoparticles in Psychotic Disorders

Psychotic disorders are clinically driven by cognitive conditions, i.e., delirium, dementia, hallucination, delusions, and schizophrenia. Conventionally available antipsychotics suffer from poor bioavailability, significant extrapyramidal effects, poor brain delivery due to the BBB, and drug-related peripheral toxicity. PNPs are under clinical trials for the management of psychotic symptoms and emerge out as more efficient and efficacious for delivery of antipsychotics to the brain (Annu Rehman et al., 2019). A rational (ligand-based PNP) approach for targeting antipsychotic drugs to the brain to overcome the limitations of conventional dosage forms is under research and discussed below.

T80-coated chitosan NPs of doxycycline hydrochloride were prepared by Yadav and associates for the treatment of psychosis. Optimized NPs were evaluated for their efficacy in the ketamine-induced psychotic mouse model. The NPs exhibited 237-nm particle and 78.16% drug entrapment efficiency. The NPs easily passed through the BBB and demonstrated significant antipsychotic activity. A marked increase in GSH and GABA levels was observed after oral administration of T80-coated doxycycline hydrochloride NPs in ketamine-induced psychotic mice. However, a marked decrease in MDA level, TNF-α, and dopamine levels was noticed in the NP-treated group compared to the toxic group. The above results indicated the enhanced penetration of doxycycline hydrochloride through the BBB with T80-coated NPs (Yadav et al., 2017).

*Author’s opinion*: The study demonstrated the potential of targeted NPs for the management of psychotic disorders and might be potential carriers in the treatment of neurological disorders.

### Polymeric Nanoparticles in Brain Tumors

GBM is the most destructive and common kind of brain tumor, affecting a large number of populations globally. Chemotherapy for the treatment of brain tumors is a challenging therapy, and the presence of the BBB also hinders the permeation of active drugs to the brain. GBM treatment is the most difficult despite having multimodal treatment facilities like radiation therapy, systemic chemotherapy, and surgical incision (Li et al., 2014). Moreover, new diagnostic strategies such as immunotherapy, new drugs, and PNPs are currently the subject of clinical trials for GBM treatment. Ligand-conjugated PNPs have shown potentiality in the treatment of GBM due to specific drug delivery at the tumor site. Targeted PNPs designed by various researchers for brain tumors treatment are described below.

Malignant brain tumors often cause alteration or disruption of the BBB, which causes an increase in heterogeneous vascular permeability throughout the tumor and its outside (Sprowls et al., 2019). The vascular delivery of therapeutics in brain tumors is hindered by the characteristics of both the BBB and the blood–brain tumor barriers (BBTB). The transport of many therapeutics and fluorescent depends on the extent of vascular permeability, as many of them reach the tumor *via* vasculature, thus limiting the extent of treatment in the region of the tumor (Belykh et al., 2020). The disruption of the BBB is a key barrier to the treatment success of brain tumors (Karmur et al., 2020).

**FIGURE 8:**
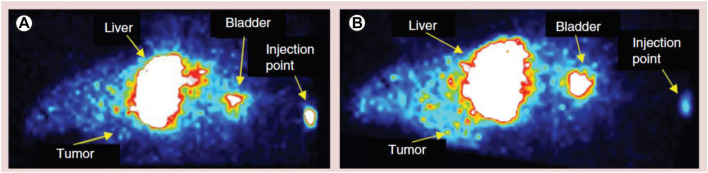
Comparative image of a tumor-bearing mouse injected with different polymeric nanoparticle formulations. **(A)** Silver-polymeric nanoparticles-^99m^Tc and **(B)** silver/alisertib-polymeric nanoparticles–chlorotoxin-^99m^Tc post-injection. Reproduced from open-access Journal under the term of Creative Commons Attribution (Locatelli et al., 2014).

Chlorotoxin (Cltx)-consolidated-alisertib/silver-(Ag)-NPs were prepared *via* nanoprecipitation using PLGA-b-PEG-COOH as copolymer. *In vitro* studies conducted in 87 MG human glioblastoma cell line revealed that with increasing concentrations of alisertib (0.001–10 μM) in Cltx-consolidated-alisertib/Ag-NPs, the cell viability decreased when compared to control (untreated cells). The cell viability was found to be 98% and 99%, respectively, after 48 and 72 h of exposure to NPs as compared to alisertib alone at each time point. Figure 8 shows the comparative analysis of Ag PNPs and Cltx/alisertib-consolidated-Ag-NPs injected in tumor-bearing mice. Nontargeted Ag-NPs-^99m^Tc showed approximately 0.6% concentration in the tumor after 60 min of injection, indicating the enhanced retention and drug permeability. Also, Cltx consolidated alisertib/Ag NPs reduced the tumor growth size, revealing the synergistic effect as compared to Cltx consolidated Ag NPs and control groups, which did not show a marked effect on the size of the tumor. The above research concluded the potentiality of these novel therapeutics for targeted delivery of therapeutics in the treatment of GBM (Locatelli et al., 2014).

GSH-conjugated PEG-PLGA NPs of DTX were prepared to enhance the brain targeting efficiency of DTX in brain tumors. *In vitro* results indicated the burst release of DTX initially from NPs for 24 h following the controlled release of DTX (18.49% ± 5.21%) for 10 days. MTT assays in C6 and RG2 cells revealed that GSH-conjugated NPs of DTX reduce the cell viability when compared to untreated cells and PEG-PLGA NPs and DTX suspension. The permeation of the drug was analyzed utilizing *in vitro* BBB (Transwell™) model. GSH-conjugated PEG-PLGA NPs showed time-dependent release of DTX in the first 16 h followed by constant release over 48 h, suggesting the ability of NPs in permeating the BBB (Grover et al., 2014).

*Author’s opinion*: The above research concluded that GSH-conjugated PEG-PLGA NPs of DTX could be a novel approach in targeting DTX to the tumor site *via* the existing transport mechanism of GSH in battling the brain tumor.

Aptamer (U2)-conjugated gold NPs were prepared for GBM therapy. U2-gold NP complex was synthesized by dissolving 5ʹ thiol-modified aptamer in phosphate buffer and then deprotecting in dark with TCEP. Aptamer and TCEP were separated using the NAP-5 column, and then U2 was purified. Excess of gold NPs was added to purified U2 and underwent 20-min reaction; then 0.1 M of phosphate buffer and sodium chloride was added and kept overnight for a reaction and then centrifuged for 30 min to remove aptamer and free gold. Cell line studies in U87-EGFRvIII depicted that U2 aptamer-conjugated gold NPs cross the BBB and reach the tumor by inhibiting the EGFR pathway and helping in preventing damage DNA repair in cells of GBM. *In vivo* study conducted in GBM-bearing mice indicated prolongation in mice survival rate with U2-conjugated gold NPs as compared to normal saline-treated mice. The above findings suggested the potential of aptamer (U2)-gold NPs as a targeted drug carrier for GBM therapy (Peng et al., 2020). Table 1 summarizes the different kinds of PNPs used for drug-targeting to the brain tumor.

**TABLE 1:** Polymeric nanoparticles for drug targeting to brain tumor.

Formulation	Outcomes	References
Camptothecin-loaded adenosine-modified NPs of polylactic acid and hyperbranched polyglycerol co-polymer	Controlled release of camptothecin, increment in BBB uptake, and enhanced brain accumulation of modified NPs were observed after intravenous administration of NPs compared to free NPs	Saucier-Sawyer et al. (2015)
Pluronic F68-coated PLGA NPs containing sodium oleate (OS) conjugate of methylene blue (MB) (MBOS)	Optimized NPs of MBOS revealed a 1.6-fold enhancement in BBB crossing than free MB and MBOS. At 10-μM concentration, NPs showed an increase in ATP levels in both U87 and T98G cell lines when compared to the control group. Also, a 1.5-fold increase in oxygen consumption rate with MBOS-NPs compared to free MB in T98G cells. The NPs showed a 25% decrease in cell viability for 144 h	Castañeda-Gill et al. (2017)
Tf-modified resveratrol encapsulated PEG-PLA NPs	*In vitro*, MTT assay showed higher cytotoxicity, enhanced cellular uptake, and reduction in tumor accumulation and volume with Tf-PEG-PLA-NPs of resveratrol followed by PEG-PLA-NPs as compared to the free drug in U87 and C6 glioma cells. Prolongation of life expectancy of rats bearing C6 glioma	Guo et al. (2013)
Tf consolidated magnetic silica PLGA NPs of doxorubicin (DOX) and paclitaxel (PTX)	*In vitro* cytotoxicity in U-87 cells revealed higher cytotoxicity in treated cells with Tf-DOX-PTX-NPs followed by DOX-PTX-NPs, DOX-PTX-NPs, and free Tf	Cui et al. (2013)
	Enhanced therapeutic efficacy of Tf-DOX-PTX-NPs *in vivo* in nude mice xenograft BALB/c and intracranial U-87 model compared to treatment with individual drug NPs with no acute toxicity	
PTX-loaded PLGA-*co*-PEG block copolymer	PTX-PLGA-PEG-loaded NPs with size of 70 nm exhibited 100-fold higher and faster diffusion than similar size PLGA-NPs of PTX. Enhanced therapeutic efficacy of PTX as PEGylated NPs of PTX delayed the tumor growth and enhanced distribution to brain tumor parenchyma	Nance et al. (2014)
Cetuximab magnetic iron-oxide nanoparticles conjugated to the EGFR inhibitor	Enhanced uptake of cetuximab FeO NPs by EGFR- as well as EGFRvIII-expressing GSCs, compared to FeO NPs alone and cetuximab alone	Hadjipanayis et al. (2010)
Curcumin-loaded CRGDS-conjugated magnetic NPs (CR-CRGDS-NPs)	*In vitro* cytotoxicity results evinced a 6-fold increase in the cytotoxic effect of magnetic-CR-CRGDS-NPs in glioblastoma cells in comparison to CR-CRGDS-NPs. The treatment with NPs in combination with radiofrequency showed stronger antitumor activity in GBM cells.	Missaoui et al. (2018a)

Note. NPs, nanoparticles; BBB, blood–brain barrier; PLGA, polylactide-*co*-glycolide; Tf, transferrin; PEG, polyethylene glycol; EGFR, epidermal growth factor receptor; GBM, glioblastoma.

### Polymeric Nanoparticles in Alzheimer’s Disease

AD is a chronic and growing neurodegenerative disorder that occurs due to the accumulation and accretion of Beta-amyloid (βA) peptides (Querfurth and LaFerla, 2010). AD is a protein conformational disease that is primarily caused by soluble protein aberrant processing and polymerization (Tiwari et al., 2019). Conferring to the UN Aging Program and US Centers for Disease Control and Prevention, it has been predicted that there will be an immense increment in the populations (above the age of 60 years) from 420 million to 1 billion by 2030 (Qiu et al., 2009; Trends, 2003; United Nations (2002). De, 2002). Moreover, the availability of present treatments is unable to cure or delay the advancement of AD, as most drugs are unable to permeate through the BBB and thus unable to reach the brain target site (Xu et al., 2020). Therefore, there becomes a need to develop NPs by incorporating the drugs to improve the targetability and therapeutic efficiency of AD drugs.

Rhynchophylline (RIN) is the main active tetracyclic oxindole alkaloid stem found to be effective in the treatment of AD, but its poor aqueous solubility, penetrability, and bioavailability limit its use in AD. Therefore, to achieve its therapeutic effect, Xu and his associates developed methoxy PEG-PLGA NP loaded with (RIN) attached with T80 (T80-NPs-RIN) using the nanoprecipitation method to enhance its brain targeting efficacy. *In vitro* BBB study was carried out among NPs and T80-NPs-RIN using mouse brain endothelial cell b.End.3, and it was observed that the uptake efficiency of T80-NPs-RIN in b.End.3 was higher when compared to that of uncoated NPs. Moreover, the incorporation of T80 in the formulation resulted in improvement in the neuroprotective outcome. Additionally, the hemolytic data revealed that T80-NPs-RIN did not instigate any hemolysis, displaying virtuous biocompatibility. Hence, the study was later concluded by depicting that the T80-coated NP had more efficiency towards the treatment of AD therapy as compared to NPs and free RIN (Xu et al., 2020).

**FIGURE 9:**
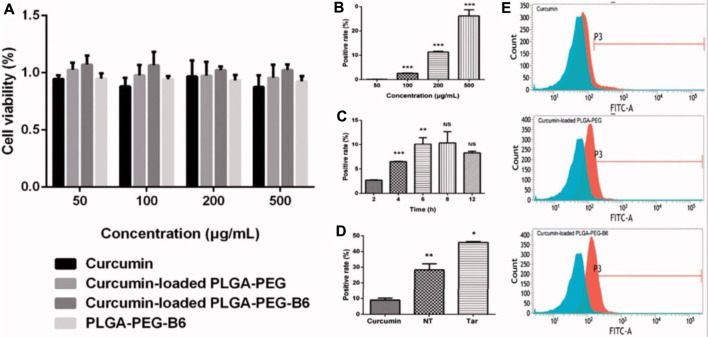
Cell viabilities and cellular uptake efficiency. **(A)** Results of cell viabilities by CCK-8 assay after 24-h incubation with concentrations of 50, 100, 200, and 500 μg/ml of Cur, Cur-loaded PLGA-PEG, Cur-loaded PLGA-PEG-B6, and PLGA-PEG-B6, respectively. Cells without treatment were regarded as the control group. Data as mean ± SD, *n* = 3. HT22 cellular uptake **(B)** 50–500 μg/ml Cur-loaded PLGA-PEG-B6 nanoparticles at 37°C incubation for 4 h. **(C)** 200 μg/ml Cur-loaded PLGA-PEG-B6 nanoparticles at 37°C incubation at different times. (D) 500 μg/ml Cur, Cur-loaded PLGA-PEG, and Cur-loaded PLGA-PEG-B6 nanoparticles at 37°C incubation for 6  h. Reproduced from open-access Journal under the term of Creative Commons Attribution. CCK-8, Cell Counting Kit-8; PLGA, polylactide-*co*-glycolide; PEG, polyethylene glycol.

Fan et al. developed an innovative brain targeting PLGA-PEG NPs loaded with curcumin (CUR) attached with B6 peptide using the ring-opening method. HT22 cells were used to perform cellular uptake study and cytotoxicity studies among the developed formulations (PLGA-PEG-B6/CUR), free CUR, uncoated formulation (PLGA-PEG/CUR), and PLGA-PEG-B6 NPs. It was observed that all the particles were compatible and exhibited a low toxicity profile as shown in Figure 9A. On the other hand, in the case of the cellular uptake study, it was observed that PLGA-PEG-B6/CUR NPs enormously enhanced their cellular uptake when compared with other formulations. The uptake effectiveness was found to be enhanced from 8.94% ± 1.10% to 45.72% ± 0.48% as shown in Figures 9B–D. Morris’s water maze parameter further demonstrated that PLGA-PEG-B6/CUR could immensely upsurge learning and memory capacity on APP/PS1 mice when compared with free CUR. Moreover, the data obtained by performing *ex vivo* parameters, for instance, immunostaining, Western blotting, and Bielschowsky’s silver staining displayed that PLGA-PEG-B6/CUR could inhibit the hippocampal βA development followed by deposition of tau-hyperphosphorylation. Therefore, it was concluded that PLGA-PEG-B6/CUR is a more promising carrier for the management of AD (Fan et al., 2018).

As per the etiology of AD, deteriorated interaction between βA with a surplus quantity of metal ions is found (Castellani et al., 2010). Thus, surpassing this disruption *via* NPs can be a better approach. Therefore, Sun et al. developed quercetin-loaded PLGA NPs (PLGA at Q) NPs *via* the double emulsion solvent evaporation technique. The developed formulation showed less cytotoxicity on SH-SY5Y cell lines in addition to inhibition and disruption assay on Aβ_42_ fibrils depicting that PLGA (at Q) NPs had more potential than any other formulation. It was further observed that there was a dose-dependent effect of the formulation on SH-SY5Y cells. Moreover, it was observed that the developed formulation had the potential to obstruct the neurotoxicity of Zn^2+^–Aβ_42_ and further increase the feasibility of neuron cells. The data obtained by performing behavioral studies using APP/PS1 mice depicted that the formulation was able to limit cognition and memory damage. No adverse effects of the formulation were reported in any of the organs after the histopathological study. Thus, the study was then concluded by stating that the prepared nanoformulation was efficient in delivering the drug at the targeted site with increased therapeutic efficacy and decreased side effects (Sun et al., 2016). Table 2 depicts the PNPs used for drug-targeting to the brain for the treatment of AD.

**TABLE 2:** PNPs for drug targeting to the brain in Alzheimer’s disease.

Formulation	Outcomes	References
Dexibuprofen (DXI)–PLGA nanosphere PEG	Considerable improvement was observed in the deposition of Aβ in addition to the activation of the glial cells and cognitive effects in the mouse model	Sánchez-López et al. (2017)
PLGA NPs of epigallocatechin-3-gallate (EGCG) and ascorbic acid (AA)	The data obtained showed that the EGCG/AA NPs were able to deliver EGCG 5-fold higher than EGCG, empty NPs	Cano et al. (2019)
Memantine PLGA NPs	NPs demonstrated a marked downfall in memory impairment in transgenic mice	Sánchez-López et al. (2018)
Pioglitazone PLGA NPs	PLGA-PEG PGZ NPs showed a marked reduction in the concentration βA localized at the cerebral cortex	Silva-Abreu et al. (2018)

Note. PNPs, polymeric nanoparticles; PLGA, polylactide-*co*-glycolide; PEG, polyethylene glycol; NPs, nanoparticles.

### Polymeric Nanoparticles in Stroke

Stroke is a clinical condition that comprises the development of disturbance in the focal signs of cerebral functions lasting for more than 24 h or causing the demise of the patient with no deceptive cause apart from those having vascular origin (Chugh, 2019). Stroke has been categorized depending upon the hurdle in the supply of blood through the blood vessels to the brain, which includes hemorrhagic stroke and ischemic stroke. Hemorrhagic stroke arises due to the falling out of blood out of the blood vessels leading to the spilling of blood in the intracranial cavity, whereas ischemic stroke happens due to the obstruction of the blood vessels that restricts the blood supply to the brain (Musuka et al., 2015). Besides, ischemic stroke covers 68% of all strokes globally than hemorrhagic stroke accounts for 32% (Lozano et al., 2012). Thus, there becomes a necessity to develop a nifty drug delivery system with the required biological and physicochemical properties including targeting ability, extended circulation time, and sustained release of drug to specific target cells.

Ischemic stroke is associated with the enhancement in reactive oxygen species (ROS) in the ischemic neuron causing the injury or death of the neurons. So Lv and associates formulated a ROS-sensitive nanocarrier to deliver the neuroprotective agent, i.e., NR2B9C at the stroke site in contrast to ischemic brain damage. The nanocarrier is comprised of a dextran polymer core altered with the boronic ester (ROS-sensitive) along with a red blood cell (RBC) membrane shell followed by insertion of the homing peptide (SHp). The developed NP (SHp-RBC-NP) showed controlled release of NR2B9C at ischemic brain tissues. Additionally, the study further revealed that the formulation possessed marked cytotoxic activity against glutamate-induced cytotoxicity in PC-12 cells. Moreover, the data obtained by performing *in vitro* and pharmacokinetics studies showed that NPs could considerably extend the systemic circulation of NR2B9C, thereby targeting the ischemic brain tissues. Therefore, the study was concluded by suggesting that the developed nanocarrier could produce effective treatment against ischemic brain damage (Lv et al., 2018).

Han and associates designed chlorotoxin (CTX)-modified PLGA NPs of Lexiscan (Lx) to improve the BBB permeability of Lx and autocatalytically augmenting stroke-targeting delivery of NPs to the ischemic microenvironment into the brain. The CTX-modified Lx NPs specifically accumulated to the ischemic brain microenvironment compared to unmodified NPs, and an increment in targeting efficiency was observed. The above findings reveal that CTX can be a promising ligand for targeted delivery of therapeutics to the ischemic brain region. The targeted delivery of NPs using CTX as a ligand could be a new approach for better management of stroke (Han et al., 2016).

Researches revealed that by the use of proangiogenic genes, ischemic stroke prognosis can be improved by improving angiogenesis at the site of injury. So hypoxia-inducible factor 1-α (HIF-1α), which has an angiogenic effect, was encapsulated to hyperbranched cationic amylopectin derivative (DMAPA-Amyp) NPs and modified with RGD peptide for directly delivering it to the endothelial cells of the brain peri-infarct site. Findings suggested higher cell uptake and good biocompatibility of RGD-DMAPA-Amyp, indicating the endocytosis of this nonviral gene vector by human cells and its safety. Studies conducted in ischemic stroke rat models demonstrated higher aggregation of RGD-DMAPA-Amyp NPs to the brain pre-infarct region and vascular endothelial cells with significant enhancement in neurological function recovery. *In vivo* results indicated significant promotion in new blood vessel formation by RGD-DMAPA-Amyp/HIF-1α-AA. The results indicated that RGD-modified NPs are the safe and promising therapeutic strategy for delivering non-viral gene vectors to the brain for ischemic stroke therapy (Deng et al., 2019).

## Toxicity Status of Nanoparticles

**FIGURE 10:**
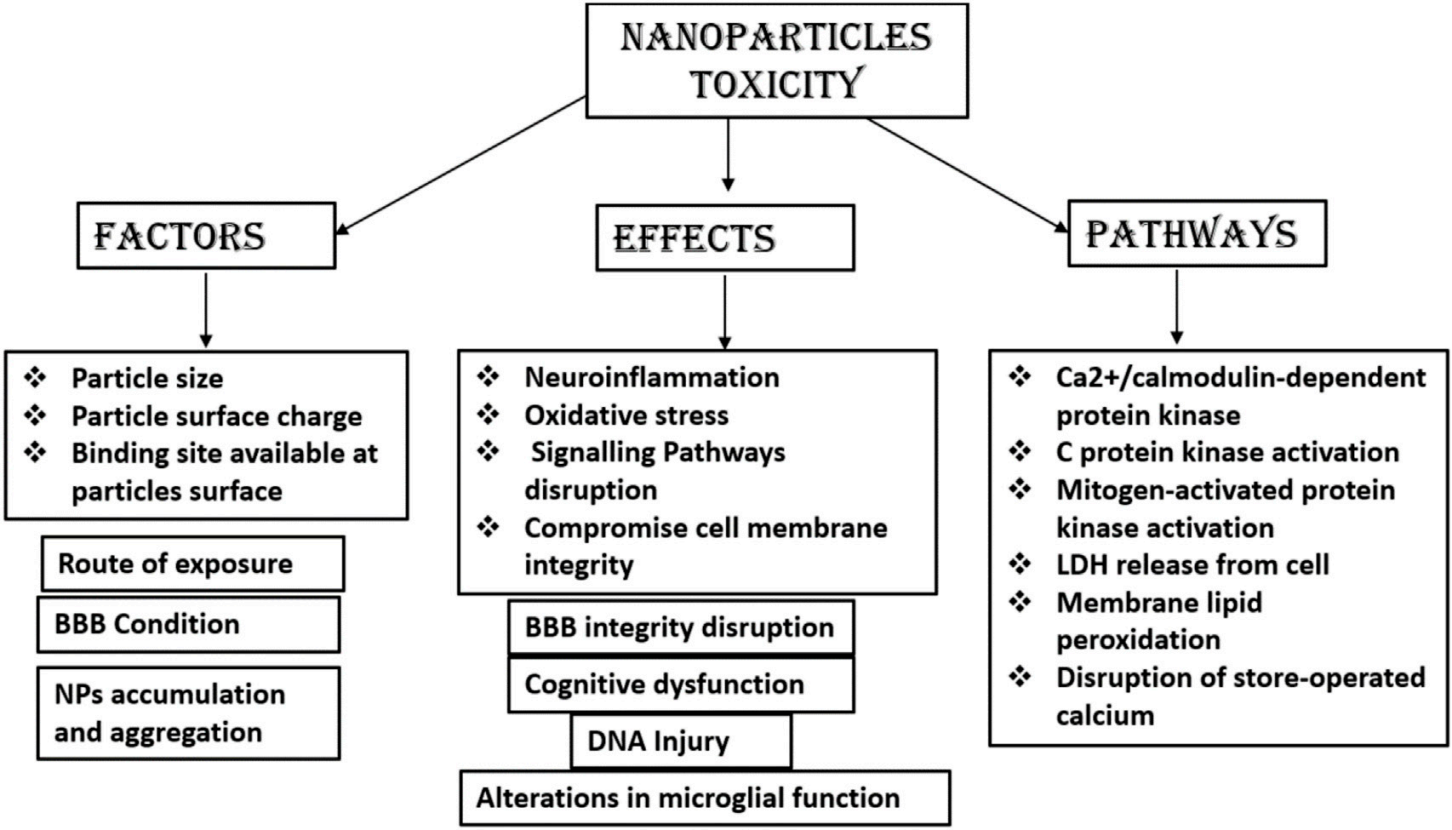
Neurotoxic effects, pathways, and factors of NPs. NPs, nanoparticles.

There is a lack of research and a lack of complete understanding of the mechanisms of NPs mediating toxicity on exposures to humans. *In vitro* and animal models have been used to detect the potential toxicity of NPs, which demonstrate neurotoxic effects of NPs, e.g., neuroinflammation and neurodegeneration in the CNS. NPs tend to accumulate in the specific brain regions after crossing the BBB and have access to neurons, astrocytes, and microglia (Leite et al., 2019). Previous research reported that NPs can induce oxidative stress, inflammation, and DNA damage and can alter the expression of genes (Leite et al., 2019). NPs having typically or relatively small size, which enter the brain by passive diffusion or by receptor-mediated endocytosis, have been shown to accumulate into the brain. Metallic NPs delivered to the brain *via* trans-synaptic transport mechanism or by disrupting the BBB results in CNS toxicity. Polysorbate 80-coated NPs of poly-*n*-butylcyano-acrylate NPs have been shown to increase interleukin-8 (IL-8) levels to disrupt the integrity of the BBB. An increase in surface area to size ratio of many NPs can result in the interaction with cellular macromolecules and result in oxidative stress (Missaoui et al., 2018b). Many metallic and carbon-based NPs have been reported as potential neurotoxic due to the increased release of cytokines, which leads to neuroinflammation resulting in neuronal death (Huang et al., 2017; Teleanu et al., 2018). However, no conclusive data are available yet on the neurotoxicity of NPs, which remains a matter of concern. Further, more research is needed to conclude the safety of NPs. Figure 10 summarizes the different factors, pathways, and effects of NP toxicity.

## Future Aspects

Rapid development has been witnessed in the formulation and development of ligand-directed based polymeric NPs in the past years for neurological disorders. It is anticipated that in the coming 10 years, a large number of ligand-based targeted PNPs will enter the phases of the clinical trials globally for the treatments of brain-related disorders, which cannot be treated properly now. The concept of PNP drug delivery vehicles concept is proposed 2 decades ago; however, there is a scarcity of translational and systemic preclinical research for drug targeting to the brain. The data obtained from different studies reviewed above indicate that PNPs could be promising vehicles for drugs targeting the brain in neurological disorders and brain tumors, as they can easily bypass the BBB and the body’s defensive mechanism, bringing sudden removal of PNPs from the circulation. Ligand-conjugated PNPs provide site-specific drug delivery, thus reducing drug-related peripheral toxicity and adverse events, reducing drug dose and dosing frequency, and improving drug therapeutic efficacy. Extensive research based on cellular and molecular levels should be done to analyze the efficacy and safety of PNPs as a whole entity. Moreover, in the future, extensive work is required to inspect the potentiality of targeted PNPs at the clinical and commercial levels. In the future, more polymers should be approved by the FDA for ascertaining their safety in brain disease, as presently very few polymers are available that are approved by the FDA. The research warrants the evaluation of the biological activity and concentration of the targeted PNPs at the target-specific site. Ligand-based NPs need to be examined carefully for their toxicity and safety in the brain. Additionally, for accurate examination of the therapeutic activity of ligand-based PNPs, *in situ* brain models, drug-resistant brain cancer models, and brain stem cell models should be established and applied to future *in vivo* studies. Further research should be conducted to ascertain the localization of PNPs to the specifically targeted cells by utilizing neuroimaging techniques, but the areas need more evaluation. The benefit–risk ratio of PNPs for clinical development should be determined. Moreover, ligand-based PNPs formulated for clinical use should be industrially scalable with remarkable therapeutic effectiveness, lower toxicity, and side effects.

## Conclusion

The current review outlines the role of ligand-conjugated PNPs for targeting the therapeutic active molecules to brain cells for the safe and effective management of neurological disorders and brain cancer. A lot of research was carried out to explore the potential of ligand-mediated PNPs for the cure of CNS disorders. *In vitro* and pharmacokinetics models manifest higher therapeutic efficacy of ligand-based PNPs compared to their conventional formulation and PNPs without ligand, with lower toxicity and side effects. However, unmet clinical trials and further research are warranted to explore the safety and efficacy of ligand-mediated PNPs.
